# Food safety related efficacy beliefs, behaviors, beliefs in myths, and the effects of educational online interventions: Data from an online survey experiment with 1,973 consumers from Norway and the UK

**DOI:** 10.1016/j.dib.2022.108102

**Published:** 2022-03-26

**Authors:** Alexander K. Koch, Dan Mønster, Julia Nafziger, Nina Veflen

**Affiliations:** a Department of Economics and Business Economics, Aarhus University, Fuglesangs Allé 4, Aarhus 8210, Denmark; b Center for Hybrid Intelligence, Aarhus University, Denmark; c Centre for Economic Policy Research (CEPR), UK; d BI Norwegian Business School, Nydalsveien 37, Oslo 0484, Norway; e Nofima, Norway

**Keywords:** Food safety, Consumers, Behaviour, Knowledge, Survey experiments, Serious games, Video-based interventions, Pre and post intervention studies

## Abstract

Data were collected in a randomized controlled trial of a game-based online intervention aimed at fostering awareness of food safety and risk-reducing behavior among consumers. 1,973 participants from the UK and Norway, aged 18–89 years, were assigned to (i) a control condition, or (ii) exposed to a brief information video, or (iii) in addition played an online game (two different conditions). In all conditions, participants answered a pre- and post-survey with seven days in between. The surveys comprised questions on sociodemographic background, preferences related to food, recent food safety behaviors and beliefs in the efficacy of a number of food safety actions, as well as beliefs in myths related to food and hygiene. Efficacy beliefs (13 questions in the pre- and post-surveys) capture how an individual thinks particular actions will affect the likelihood of contracting food-borne disease. Beliefs in myths (8 questions in the pre- and post-surveys) refer to commonly held ‘true-or-false’ beliefs with no base in scientific facts. Target behavior (21 questions in the pre- and post-surveys) refer to self-reported food safety behaviors that were targeted in the interventions. Additional questions address beliefs and behavior in relevant food safety areas that were not targeted in the interventions. The survey items related to beliefs and behaviors were based on or inspired by previous work of the SafeConsume EU consortium (www.safeconsume.eu). In the information condition, participants watched a two-minute information video about food safety. Participants were given information about five broad themes: personal hygiene (hand washing), kitchen hygiene (cleaning utensils and surfaces), washing fresh vegetables and fruits, *not* rinsing meat or poultry, checking the temperature of cooked meat or poultry. In the game conditions, participants first watched an information video (either the neutrally framed one from the information condition or a version with pictures framed to trigger a disgust reaction). Then participants prepared four recipes in an online game, where they were repeatedly confronted with food safety related actions. After each recipe, participants received feedback on how they handled a number of important food safety actions. Our survey measures provide scholars and practitioners with data from adults in Norway and the UK to perform analyses regarding consumers’ knowledge and behavior related to food safety. Data and the replication code for the associated research article Koch et al. [3] are accessible at Koch et al. [4].

## Specifications Table

**Table:** 

Subject	Food Science: Food Microbiology
Specific subject area	Data were collected in a randomized controlled trial of a game-based online intervention aimed at fostering awareness of food safety and risk-reducing behavior among consumers. The dataset includes pre- and post-intervention survey measures of food safety related beliefs and behaviors and additional variables on the background characteristics of participants.
Type of data	Survey data
How the data were acquired	Online survey, administered through the survey company Kantar/Gallup. The survey questions are reproduced in the data repository [4].
Data format	Raw data, comma separated (csv) file. Raw data, Stata dta file. Description of the variables and the survey questions, Excel file. Questionnaire text, pdf file Link: https://zenodo.org/record/6337094[4]
Description of data collection	Participants were assigned to one of four conditions (between-subject design): *Control, Info, Game*, and *DisgustGame*. Data were collected by Kantar/Gallup in Norway and the UK between December 2020 and March 2021. Inclusion criteria: Participants prepared at least two warm lunches/dinners with meat or poultry per week on average. Participants were required to use a desktop computer or notebook to access the study. The sample was stratified by gender.
Data source location	Country: Norway and UK
Data accessibility	Koch et al. [4] Repository name: Zenodo Data identification number: 10.5281/zenodo.6337094 Direct URL to data: https://zenodo.org/record/6337094
Related research article	Koch et al. [3]: Koch, Alexander K., Dan Mønster, Julia Nafziger, and Nina Veflen, Fostering safe food handling among consumers: Causal evidence on game- and video-based online interventions,” *Food Control*, 2022, *135*, 108825. https://www.sciencedirect.com/science/article/pii/S0956713522000184.

## Value of the Data

• The dataset contains information on food safety related beliefs and behaviors of 1,973 consumers in Norway and the UK in addition to several variables on background characteristics that allow further study of issues related to consumer beliefs and behavior in the field of food safety.
• Unlike many studies on game-based interventions that are run in educational contexts, the data cover adults aged 18-89 years.
• The data contain pre- and post-intervention measures for video-based and game-based educational online interventions, allowing for further analyses comparing these intervention types.
• Scholars and practitioners interested in consumer knowledge and behavior related to food safety can benefit from these data.
• The data related to our educational interventions can inform the design of future interventions and can be useful for power analyses for future studies.

## 1 Data Description

• **SafeconsumeVariables.xlsx** The file contains a description of the variables in the dataset.
• **SafeconsumeUKNOData.dta** The file contains all the raw data from the surveys administered in the study in Stata dta format (see SafeconsumeUKNOData.csv for further description).
• **SafeconsumeUKNOData.csv** The file contains all the raw data from the surveys administered in the study in comma separated format. The key variables for the study are based on questions through which we elicited beliefs in the efficacy of certain food safety actions, as well as beliefs in myths, and measured food safety related behavior.
• The data repository additionally contains the file **Safeconsumereplicationcode.do**. The file contains the code and explanations for replicating the analyses in [3] in Stata. These analyses are not discussed in the present paper.

### 1.1 Food safety related efficacy beliefs

*Efficacy beliefs* refer to an individual’s belief that a particular action will affect the likelihood of contracting food-borne disease. 20 questions in the pre- and post-surveys measured these beliefs (see Table 1). A composite measure of food safety related efficacy beliefs can be constructed from the data by summing the scores for the items 1-20, reverse coding items marked with R (recoded score=6-score) and coding the absolute distance from the ‘center’ for items marked with C (recoded score=|3-score|). The interpretation is that an increase in the recoded individual item or the composite score indicates an improvement in beliefs. Similarly, a composite measure of efficacy beliefs *targeted* by our interventions can be constructed from the data by summing the scores for the items 1-13, and a composite measure of *non-targeted* efficacy beliefs by summing the scores for the items 14-20. See below for how to construct standardized measures that are comparable across the different categories.

**Table 1:** Items in “Efficacy beliefs”.

Description

**Targeted efficacy beliefs**
Directly targeted
1. (R) Peeling unwashed vegetables/fruit
2. Rinsing unwashed vegetables/fruit
3. Picking up within 5 seconds any food that has fallen to the ground
4. (R) Heating hamburger meat such that only the inside is pink
5. (R) Cooking chicken to an inside temperature of 63 degrees Celsius
6. (R) Rinsing a whole chicken before preparation
7. (R) Rinsing hands under running water without using soap
8. Washing hands with soap under running water
9. Washing cutting boards and kitchen tools in between preparing different food items
10. Rinsing a whole melon
11. (R) Cooking an egg until soft-boiled (that is, the white is firm and the yolk is soft)${}^{*}$
Indirectly targeted
12. (R) Checking whether a food item smells fine
13. (R) Checking with a fork whether the chicken is well done

**Non-targeted efficacy beliefs**
14. (C) Using brown eggs rather than white eggs
15. (C) Only eating organic food
16. (C) Only eating home grown food
17. (C) Only eating food produced in [the UK/Norway]
18. (C) Drinking a small amount of alcohol with a meal
19. (C) Switching to a vegetarian diet
20. (R) Only eating raw food

Scale: Increases risk by a (1) large (2) small amount, Has no effect on risk (3), Decreases risk by a (3) small (4) large amount. ${}^{*}$ Targeted only in the video. R: reverse code as *recoded score*=6-*score*. C: recode absolute distance from the ‘center’ as *recoded score=|3-score|*.

### 1.2 Beliefs in myths related to food safety

A set of 8 questions in the pre- and post-surveys capture commonly held ‘true-or-false’ beliefs with no base in scientific facts, to which we refer to as *beliefs in myths* (see Table 2). These questions were based on a database of myths that the SafeConsume EU consortium collected across Europe. From this data base we selected the eight myths that were most relevant to the targeted beliefs and behaviors in the interventions. A composite measure can be constructed by summing the items and standardizing, as described below. All items need to be reverse coded to get the interpretation that a higher score is an improvement in beliefs.

**Table 2:** Items in “Beliefs in myths.

Description
**Targeted beliefs in myths**
1. (R) Fruit and vegetables that will be peeled don’t have to be washed
2. (R) Any food that has fallen to the floor, and did not stay there longer than 5 seconds, is still edible
3. (R) Only poultry, not other meats, need to be well-done to be safe to eat

**Non-targeted beliefs in myths**
4. (R) Washing your kitchen too often creates a sterile environment that is bad for building up a good immune system
5. (R) A small amount of alcohol is good to avoid food poisoning
6. (R) If the food smells and taste fine it is safe to eat
7. (R) Eggs with brown shells are safer than eggs with white shells
8. (R) Vegetarians don’t get food poisoning

Scale: Agree with statement: Yes (1) No (2). R: reverse code as *recoded score=2-score*

### 1.3 Food safety related behavior

21 questions in the pre- and post-surveys capture self-reported food safety behaviors that were targeted in the intervention (see Table 3). To construct a composite measure of *target behavior*, one needs to take into account that the questions are not all on the same scale. The procedure is instead to

1. standardize each individual item using the pre-treatment mean and standard deviation (see below)
2. take the average over the standardized scores: *(sum of the standardized scores)/21*.

**Table 3:** Items in Targeted behavior.

Description
**Targeted behavior 1-3** (Scale 1)
1. Did you wash your hands with soap?
2. Did you clean the kitchen surface?${}^{*}$
3. Did you rinse a piece of raw meat?

**Targeted behavior 4-5** (Scale 2)
4. I used a food thermometer
5. (R1) I did not check whether the meat is done

**Targeted behavior 6-21** (Scale 3)
6. (R2) A whole raw chicken
7. (R2) Raw chicken breasts
8. Raw beef
9. A whole lettuce
10. A whole watermelon
11. An apple
12. A mango
13. An eggplant
14. An onion
15. String beans
16. Brussels sprouts
17. Potatoes
18. Carrots
19. Berries
20. An avocado
21. Bean sprouts

Scale 1: Never (1), Once (2), Twice (3), 3-4 times (4), 5 times or more (5). Scale 2: Yes (1), No (2). Scale 3: How likely would you be to rinse before further preparation/consumption? No chance or almost no chance (1 in 100) (1) ⋯ Certain or practically certain (99 in 100) (11). ${}^{*}$ The behavior question “Did you clean the kitchen surface?” was accidentally omitted by the survey company and this was only noticed half-way into the data collection. Hence, it is not available for all participants. R1: reverse code as *recoded score=2-score* R2: reverse code as *recoded score=11-score*.

### 1.4 Standardization

The following procedure permits a standardized comparison of pre- and post-measures across the different categories: Standardize the individual or composite measures for the pre- and post-surveys by subtracting the mean of the corresponding pre-survey measure and dividing by the standard deviation of the pre-survey measure. The pre-post change in the standardized measure thus has the interpretation of an effect size (it says by how many pre-intervention standard deviations the measure is moved pre-post intervention) and it is comparable across the different types of measures even if they are measured on different scales.

### 1.5 Other variables

Further, the data contain additional individual characteristics, such as sociodemographic background and information on experience with cooking and food safety (see Table 4).

**Table 4:** Individual characteristics.

Description
1. Age
2. Gender (male, female)
3. Whether the participant lives in a single-person household
4. Highest level of education (Primary school, High-school/Tertiary education, University, Postgraduate)
5. Household income
6. How often the participant prepares a warm lunch or dinner with meat (including poultry) on average
7. Disgust sensitivity measured by the 7-item food disgust picture scale of [1]
8. Frequency of playing computer games
9. Whether the participant has ever worked in the food industry or in gastronomy/food service
10. Whether the participant has ever worked as a health professional
(health worker, nurse, doctor, physician, nutritionist,...)
11. Whether the participant has ever had food poisoning
12. Risk tolerance measured by the question of [2]
13. Number of children (0,1,2,3, 3 or more)
14. How often the participant felt stressed when cooking because of time pressure
15. Food-related risk tolerance: Are you a person who is concerned about getting sick from food poisoning or are you
not concerned about getting sick from food poisoning? Scale: 0: not at all concerned about getting sick...
10: very concerned about getting sick
16. Preference for eating hamburger meat pink inside rather than well done, measured by a question showing two
different hamburgers (A: pink inside, B: well done).
17. Importance of the meal being prepared under hygienic circumstances${}^{*}$
18. Importance of the meal being fast to prepare${}^{*}$
19. Importance of not messing up the kitchen when cooking${}^{*}$
20. Importance of avoiding food waste${}^{*}$

${}^{*}$ What is important when shopping for, preparing, and cooking a meal: Scale: Not important (1), Low importance (2), Neutral (3), Slightly important (4), Very important (5).

## 2 Experimental Design, Materials and Methods

The study had three parts: A pre-survey, the intervention part (watching an information video and playing a game or just watching the video), and a post-survey seven days after the pre-survey. In a between-subject design, participants were assigned to one of four conditions: Control, Info, Game, and DisgustGame. Specifically, three types of interventions were evaluated: a brief information video on food safety (*Info*), the video followed by a computerized home cooking game (*Game*), the game with a different variant of the information video that used a disgust frame (*DisgustGame*).

### 2.1 Pre-survey

All participants answered a pre-survey (see [4] for the questionnaire). Questions addressed some recent food safety related behaviors (see Table 3) and elicited beliefs in the efficacy of certain food safety actions (see Table 1), as well as beliefs in myths (see Table 2). In addition, we collected some information on sociodemographic background and preferences related to food and hygiene (see Table 4).

### 2.2 Information video

Participants in the three conditions with an intervention (*Info, Game*, and *DisgustGame*) watched a two-minute information video about food safety after completing the survey. The information video addressed five broad categories related to food safety in a home cooking setting: (1) personal hygiene (hand washing), (2) kitchen hygiene (cleaning utensils and surfaces), (3) washing fresh vegetables and fruits, (4) *not* rinsing meat or poultry, and (5) cooking foods thoroughly. The video showed simple animated graphics that were accompanied by brief statements presented both by a narrator (audio) and as text in the video. All statements were identical in the two versions of the video. The video in the *DisgustGame* differed from the video used in *Info* and *Game* by replacing some of the images used for the animations to induce a disgust reaction. For example, it showed rotten fruit or a person vomiting after eating dropped food. Except for the first statement, which introduced the overall topic of safe food handling, all of the statements were paired, such that one statement is an *important food safety action* (IFSA) (e.g., wash vegetables and fruit even if you peel them) coupled with at least one other statement which is either a fact to be aware of (e.g., vegetables and fruit can be covered with harmful bacteria) or an explanation (e.g., this avoids spreading bacteria from the peel).

The videos contained a total of 18 statements, consisting of 9 IFSA statements, 6 facts and 3 explanations, all of which conform to the syntax [Fact], IFSA, [IFSA], [Explanation], where the square brackets indicate that the order or number of entities might differ. However, an IFSA was always either preceded by a Fact or followed by an Explanation, or both. The actual sequences were (frequencies in parenthesis): Fact, IFSA (2); Fact, IFSA, IFSA (2); Fact, IFSA, Explanation (1); and IFSA, Explanation (2). The transcript of the videos is available in the supplementary materials. The videos are available as supplementary materials to the article Koch et al. [3].

### 2.3 Online game

After watching the information video, participants in the *Game* and *DisgustGame* and conditions were directed to an online game (the game can be played at https://safeconsume.eu/tools/safeconsume-game). The game setting was a household kitchen. It had a worktop with a sink, where players could wash their hands (optionally using soap) and do dishes (optionally using dish washing liquid). Players also had access to a surface cleaner and paper towels and a rubbish bin. While preparing food, players used a cutting board and a knife, a pan on the stove, and a food thermometer. The task of participants was to prepare recipes with chicken, a raw vegetable or fruit, and bread. Specifically, they were required to take the respective ingredients and prepare each of them as follows. Meat and fruit/vegetables had to be taken from a refrigerator and bread from a basket. Each food item had to be cut on a cutting board. Meat required the further step of heating it in the pan. All food items had to be served on a plate. Participants had the opportunity to engage in several types of additional behavior. They could wash their hands with or without soap, they could rinse food items, they could clean the cutting board and knife by placing them in the sink and doing washing up with or without dish washing liquid, they could measure the temperature of the chicken in the pan with a food thermometer, they could clean surfaces with a surface cleaner and kitchen towels, and they could throw into a rubbish bin food that had dropped to the ground. Once all food items were placed on the plate, they could be served with the click of a button. Participants could then leave the kitchen by pressing a button. This gave them the chance to, for example, clean up in the kitchen even after the meal was served. Leaving the kitchen completed the recipe and the participants then received feedback on how well they performed in terms of a number of *important food safety actions* (IFSAs) related to the food safety advice given in the information video:

1. Washing hands with soap before starting to cook and after preparing a food item.
2. Cleaning food preparation tools with water and dish liquid after preparing a food item.
3. Cleaning kitchen surfaces after preparing a food item.
4. Checking with a food thermometer that the chicken has an internal temperature of 74 °C before removing it from the pan.
5. Rinsing fruit/vegetables (even if later peeled) before preparing them.
6. Not rinsing raw meat.
7. Not consuming food items that dropped to the ground. (only relevant in levels 2 and 3, where the bread drops to the floor when trying to place it on the cutting board)

Participants were required to complete four recipes (called *levels*). When entering the first level, participants were shown a video tutorial that explained how to play the game with a commented play-though of a complete recipe with statements presented both by a narrator (audio) and as text in the video (the video can be seen when starting the game available at https://safeconsume.eu/tools/safeconsume-game). To reinforce the messages from the information video, in the play-through all the IFSAs were correctly performed. In levels 2-4, a clock appeared to add time pressure for participants to complete the recipe within 5 minutes. If the timer expired it turned red, but the game was not stopped. Participants received feedback whether they completed the level within the time limit or not. In levels 2 and 3, the bread dropped to the floor when trying to place it on the cutting board. Correct handling required throwing the bread into the rubbish bin and getting a new bread from the basket. In levels 2 and 4, a cat disturbed the cooking process. The participant could remove the cat from the counter by clicking on it. As long as the cat was not removed, it kept miaowing and walking over the worktop, leaving a trail of cat hair behind.

### 2.4 Post-survey

All participants answered a post-survey (see [4] for the questionnaire). Here we again asked the questions related to recent food safety related behaviors (see Table 3), elicited beliefs in the efficacy of certain food safety actions (see Table 1), as well as beliefs in myths (see Table 2).

### 2.5 Data collection

Data were collected by Kantar/Gallup in Norway and the UK between December 2020 and March 2021. Eligibility for the study was checked with a question on how often a participant prepared warm lunches/dinners with meat or poultry per week on average. Only those who answerded “at least two” were admitted to the study. Further, it was checked that participants used a desktop computer or notebook to access the study. The reason for this restriction was that the online game used unity and ran in browser and could not be played using a device with IOS or Android operating systems. The sample was stratified by gender. Participants received a fixed compensation for completing part 1 of the study and a bonus for completing part 2 (the post-survey). The exact amounts are confidential information not disclosed by Kantar/Gallup, but they should lie above to the industry standard for simple surveys because participants were given a bonus to complete both parts of the study.

The median duration for part 1 of the study was 15 min. for *Control*, 18 min. for *Info*, 65 min. for *Game*, and 61 min. for *DisgustGame*. Part 2 (the post-survey) had a median duration of 9 min.

## Ethics Statements

As a low risk study on human behavior, the study was exempted from ethics review by the Health Research Authority in the UK, by the Norwegian Centre for Research Data, and Nofima’s ethical board in Norway. Participants gave informed consent.

## CRediT authorship contribution statement

**Alexander K. Koch:** Project administration, Conceptualization, Methodology, Formal analysis, Writing – original draft, Writing – review & editing. **Dan Mønster:** Project administration, Conceptualization, Writing – review & editing. **Julia Nafziger:** Conceptualization, Methodology, Writing – review & editing. **Nina Veflen:** Funding acquisition, Conceptualization, Writing – review & editing.

## Declaration of Competing Interest

The authors declare that they have no known competing financial interests or personal relationships that could have appeared to influence the work reported in this paper.

## Data Availability

Food safety-related efficacy beliefs, behaviors, beliefs in myths, and the effects of educational online interventions: Data from an online survey experiment with 1,973 consumers from Norway and the U (Research Data) (Zenodo).

## Supplementary materials

Image, application 1
