# Supplementary material for: Food safety related efficacy beliefs, behaviors, beliefs in myths, and the effects of educational online interventions: Data from an online survey experiment with 1,973 consumers from Norway and the UK
Source: Data Brief. 2022 Mar 26;42:108102. doi: 10.1016/j.dib.2022.108102 (PMC9006754; doi:10.1016/j.dib.2022.108102)
Supplement: Supplementary file 1 [file mmc1.pdf]

Online Supplement for
Food safety related efficacy beliefs, behaviors, beliefs in myths, and the effects of educational online interventions: Data from an online survey experiment with 1,973 consumers from Norway and the UK.

Alexander K. Koch[a,b,*], Dan Mønster[a], Julia Nafziger[a,c] & Nina Veflen[d,e*]

[a]Department of Economics and Business Economics, Aarhus University,

Fuglesangs Allé 4, 8210 Aarhus, Denmark

[b]Center for Hybrid Intelligence, Aarhus University, Denmark

[c]Centre for Economic Policy Research (CEPR), UK

[d]BI Norwegian Business School, Nydalsveien 37, 0484 Oslo, Norway

[e]Nofima, Norway

**Corresponding author: A. Koch, Phone: +4587165539, Email: akoch@econ.au.dk.

S.1 Information videos

There are two versions of the information videos. Both versions have the same statements, reproduced in Table S.1 and only differ in the animated images used. The videos are available as supplementary materials to the article Koch, Alexander K., Dan Mønster, Julia Nafziger, and Nina Veflen, "Fostering safe food handling among consumers: Causal evidence on game- and video-based online interventions," *Food Control*, 2022, *135*, 108825. https://www.sciencedirect.com/science/article/pii/S0956713522000184.

Table S.1: Transcript of the information videos

Sequence	Category	Text
1a	Introduction	Unsafe handling and preparation of food in your kitchen can cause illness.
1b	Introduction	Here is what you can do to avoid getting sick.
2a	Fact	Your hands often pick up bacteria that can cause illness.
2b	IFSA	Wash hands with soap and water, and dry them before and after handling a food item.
3a	Fact	Vegetables and fruit can be covered with harmful bacteria.
3b	IFSA	Wash them even if you peel them.
3c	Explanation	This avoids spreading bacteria from the peel.
4a	Fact	Washing poultry or meat can spread harmful bacteria through water droplets.
4b	IFSA	So do not wash raw poultry or meat.
5a	Fact	Bacteria from raw or undercooked eggs, poultry and meat can cause illness.
5b	IFSA	Use a food thermometer to check that poultry and meat are done.
5c	IFSA	Heat eggs until yolk and white are firm.
6a	IFSA	Clean the kitchen surface with surface cleaner and a paper towel after each food item you handle.
6b	Explanation	This removes harmful bacteria.
7a	IFSA	Wash and scrub kitchen utensils with detergent after each use.
7b	Explanation	This avoids spreading harmful bacteria from one food item to another.
8a	Fact	When food falls on the ground it can catch harmful bacteria.
8b	IFSA	Wash any food that has touched the ground.
8c	IFSA	... or throw dropped food out if it cannot be washed.

IFSA: Important food safety action.
